# Validating and prioritizing key indicators for blended MOOC implementation in English language learning using the fuzzy Delphi method

**DOI:** 10.1016/j.mex.2025.103668

**Published:** 2025-10-09

**Authors:** Anh Tuan Pham, Muhammad Ridhuan Tony Lim Abdullah

**Affiliations:** a Department of Management, Universiti Teknologi PETRONAS, Perak, Malaysia; b Department of English, FPT University, Can Tho, Vietnam

**Keywords:** Blended learning, Massive Open Online Courses, Indicators, EFL instruction, Fuzzy Delphi analysis

## Abstract

This study presents a structured method for validating and prioritizing key indicators necessary for implementing blended Massive Open Online Courses (bMOOCs) in English language learning, using the Fuzzy Delphi Method (FDM). Despite the growing adoption of MOOCs in higher education, especially during and after the COVID-19 pandemic, a lack of standardized criteria for evaluating blended learning in English as a Foreign Language (EFL) contexts persists. To address this gap, this method offers a systematic approach that captures expert consensus through fuzzy logic, enhancing decision-making under uncertainty. A panel of 15 experts evaluated 21 proposed sub-indicators across six domains. The FDM process included systematic steps, resulting in a validated set of 20 sub-indicators.

• A comprehensive 8-step FDM process was used to validate 20 key sub-indicators for bMOOC implementation in English learning.

• Indicators were categorized into six domains: learner analysis, objectives, materials and technology, teaching methodologies, learner participation, and assessment.

• The method enhances transparency and replicability in developing evaluation frameworks for blended MOOC environments.

This validated indicator set provides a foundation for instructional design, policy planning, and quality assurance in EFL programs adopting blended MOOC models.

## Graphical abstract

**Figure:**
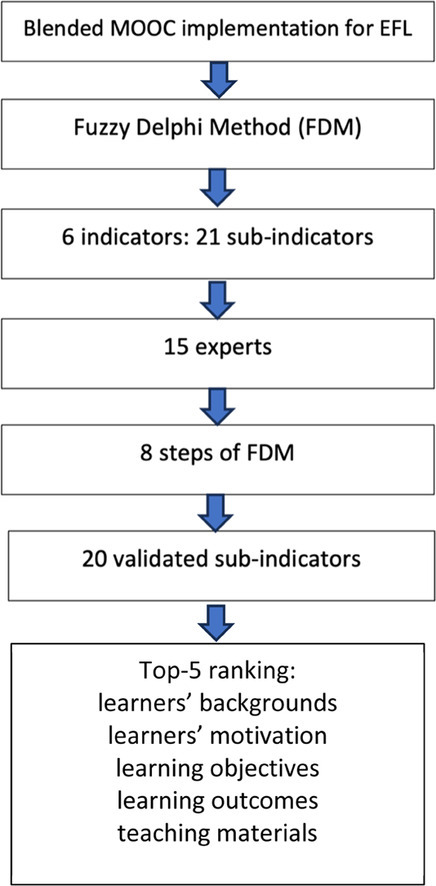

## Specifications table

**Table:** 

**Subject area**	
**More specific subject area**	Educational psychology, Educational technology, EFL education
**Name of your method**	Fuzzy Delphi Method
**Name and reference of original method**	Kaufmann, A., & Gupta, M. M. (1988). *Fuzzy mathematical models in engineering and management science*. North-Holland.
**Resource availability**	Questionnaire and fuzzy computation template are available upon request. Google Forms was used for expert data collection; Microsoft Excel was used for fuzzy calculations. Dataset available at: https://doi.org/10.5281/zenodo.17257906

## Background

The rise of online education has been largely driven by advancements in computer and internet technologies [1]. While widely adopted in developed countries, such innovations remain limited in developing regions, mainly due to financial and infrastructural challenges [2]. High-quality online courses are often offered by prestigious institutions like Harvard and Oxford, where robust pedagogical strategies ensure sustainability and excellence in education [3]. For developing countries, narrowing this quality gap is crucial [2].

The introduction of Massive Open Online Courses (MOOCs) by George Siemens and Stephen Downes in 2008 revolutionized the online learning landscape [4]. The COVID-19 pandemic further accelerated this shift, prompting a global transition to remote education [5]. Today, MOOCs serve over 220 million users through nearly 20,000 courses offered by over 950 universities worldwide [4], making them a powerful medium for distance learning. Their global appeal lies in offering flexible, high-quality content to a wide audience [6]. However, as educational institutions transition back to on-campus instruction, many are adopting blended approaches that combine face-to-face teaching with online learning to reduce contact time and increase flexibility [7,8]. Traditional MOOCs, though beneficial, have faced criticism for their teacher-centered design and limited interactivity. To address these shortcomings, educators are increasingly integrating MOOCs into physical classrooms, a model known as blended MOOCs (bMOOCs) [9,10].

Blended MOOCs have become an emerging trend in higher education, as they aim to combine the accessibility of online learning with the interactive benefits of traditional instruction [11]. Research indicates that such integration can enhance student performance and academic outcomes [12]. In English as a Foreign Language (EFL) instruction, MOOCs provide flexible learning opportunities that compensate for limited in-person class time [[13], [14], [15]], making them effective tools for language development [16,17]. Blended learning models not only improve learning outcomes but also promote learner autonomy [[18], [19], [20]]. In countries like Russia and China, bMOOCs have been linked to improved language proficiency and increased learner engagement [[21], [22], [23]], particularly when MOOCs are integrated into existing classroom instruction. In Russia, for example, Zubkov [21] found that incorporating MOOC materials into English courses for technical students enhanced their vocabulary acquisition and communication skills in domain-specific contexts. Similarly, in China, studies such as Wang et al [17] have shown that blended learning environments combining online MOOC content with face-to-face guidance significantly improved students’ reading, listening, and speaking abilities, while also fostering greater motivation and participation in learning activities. These outcomes suggest that bMOOCs, when thoughtfully implemented, can support both linguistic development and active learner involvement in EFL education. Overall, students have expressed positive attitudes toward integrating MOOCs into English language education [24].

Despite these advancements, a major concern persists: the lack of clear, standardized indicators to evaluate the effectiveness of bMOOCs in EFL contexts. While studies have explored factors such as engagement, content quality, and assessment methods, these elements are not uniformly defined or measured across English language programs. This inconsistency hampers the development of effective teaching strategies and complicates assessment practices. To bridge this gap, it is essential to establish and validate a comprehensive set of indicators tailored to the needs of EFL learners in bMOOC settings. This will not only strengthen instructional design but also enhance the learning experience and academic success of students participating in blended English language programs. To bridge theoretical understanding and practical implementation, the following section presents the methodological design that guided this research.

## Method details

This study employed the Fuzzy Delphi Method (FDM) due to its strength in managing ambiguity and subjective opinions while facilitating consensus among experts. Recognized for its utility in generating and refining ideas, FDM is particularly effective when addressing complex or context-specific issues. Initially introduced by Kaufmann and Gupta [25], the method integrates principles from fuzzy set theory and the classic Delphi technique [26]. By merging the two, FDM offers a structured, data-informed approach for expert decision-making.

The traditional Delphi method involves repeated rounds of expert surveys to arrive at a collective judgment on a given issue [27]. FDM enhances this by incorporating fuzzy logic, which allows for more nuanced assessments rather than binary choices. This enhances the sensitivity of the analysis and minimizes information loss during aggregation. Additionally, FDM requires fewer rounds than classical Delphi, reducing response fatigue while still achieving a strong consensus. FDM is also more appropriate for validating non-hierarchical constructs such as pedagogical indicators. Compared to the Analytic Hierarchy Process (AHP), FDM requires fewer comparisons and is less cognitively demanding, making it more practical for educational research contexts. Therefore, the FDM was considered the most efficient and conceptually suitable method for prioritizing key indicators in bMOOC implementation. The FDM process consists of eight structured steps (see Fig. 1): questionnaire design and delivery, expert panel selection, linguistic scale and fuzzy numbers, data collection and averaging, threshold value and consensus, aggregating fuzzy evaluations, defuzzification, and final ranking.

**Fig. 1:**
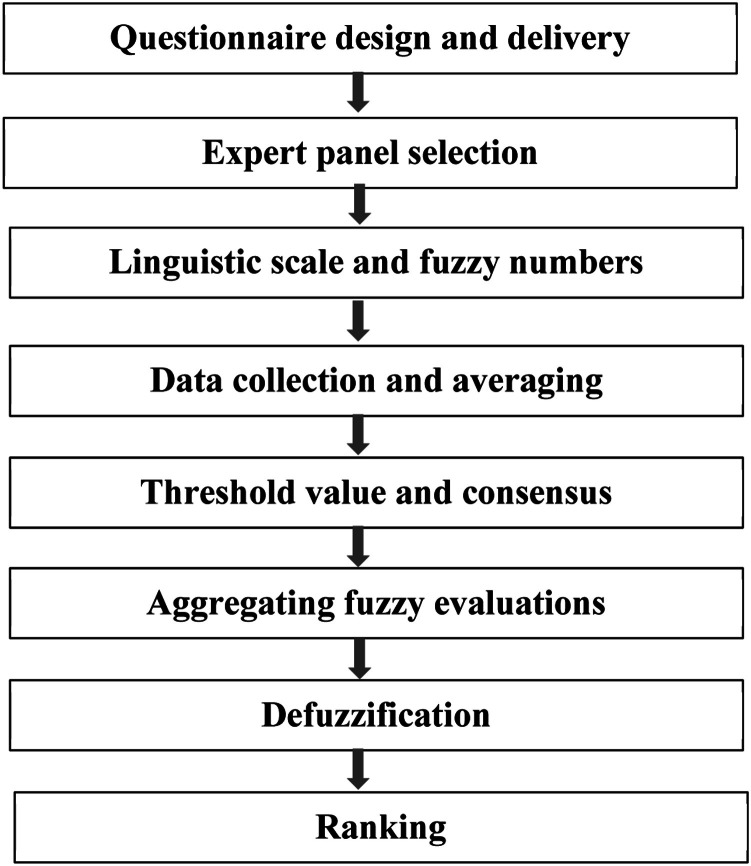
The process of the fuzzy Delphi method.

### Step 1: questionnaire design and delivery

The primary aim of the questionnaire was to identify and prioritize key indicators necessary for designing and delivering bMOOCs in English language learning. The development process began by drafting a list of sub-indicators based on a literature review. Each sub-indicator was written clearly and concisely to reflect the context of bMOOCs for English learning. The development of the questionnaire is as follows:

- Drafting the questionnaire:

The list of indicators and sub-indicators was used to create the first version of the questionnaire. Each sub-indicator was written as a clear statement to make it easy for experts to understand. Twenty sub-indicators were extracted from the literature relevant to the context of blended MOOCs for English learning.

- Expert review:

The draft was then reviewed by a group of professionals with experience in blended learning, English language teaching, MOOCs, and instructional design. Their feedback helped improve the wording, remove repeated sub-indicators, and make sure all key areas were covered. After that, the revised questionnaire was tested by 15 experts, who were asked to judge whether each sub-indicator was useful by answering “Yes” or “No.”

- Final version and distribution:

Based on expert suggestions, one new sub-indicator (SI15)—"learners' participation in offline sessions"—was added. The final questionnaire included 21 sub-indicators grouped into six categories: analysis of learners, objectives, course materials and technology, teaching methodologies, learner participation, and feedback and assessment. It was shared with the expert panel using Google Forms to make it easy to complete. Experts rated each sub-indicator’s importance using a 7-point scale, where 1 for "least important" and 7 for "extremely important." The list of sub-indicators is presented in Table 1.

*Step 2: Expert Panel Selection*

**Table 1:** List of proposed indicators and sub-indicators for blended MOOC implementation for EFL learning.

Indicators	Sub-indicators (SI)
Analysis of learners	1. “Clearly defined learners’ backgrounds (age, learning styles, and current level of English proficiency)”
	2. “Learners’ familiarity with MOOC platforms”
	3. “Learners’ motivation and goals to learn English”
Objectives	4. “Clearly defined and measurable learning objectives”
	5. “Achievable learning outcomes within a time frame”
	6. “The alignment of learning outcomes with students’ needs and levels”
Course materials and technology	7. “Availability and accessibility to MOOCs (e.g. LMS, online materials, social blogs, etc.)”
	8. “The suitability of teaching materials (textbooks, videos, quizzes, etc.)”
	9. “Variety of teaching content that develops English language skills”
Teaching methodologies	10. “Appropriate proportion of online (self-paced) activities and offline mentoring activities”
	11. “Suitability of English language teaching methodologies”
	12. “Effectiveness of using teaching aids (visual aids, multimedia, instructional materials)”
	13. “Availability of effective learning support (technical issues, assignment issues, etc.)”
Learners’ participation	14. “Level of interaction between students and other peers, instructors”
	15. “Learners’ participation in offline mentoring sessions”
	16. “Effective collaboration in interactive activities”
	17. “Effective self-regulated learning in online and offline English classrooms”
Feedback and assessment	18. “Appropriate assessment methods (formative and summative assessment)”
	19. “Instructor, peer feedback and online auto-feedback on student progress in specific English skills”
	20. “The revision of course syllabi based on feedback and performance data”
	21. “The offline final examinations to re-evaluate students’ English performance”

To ensure the credibility of results, 15 experts were carefully selected. These participants came from diverse backgrounds relevant to bMOOC development for English language instruction. The panel included university administrators (02), lecturers (03), curriculum designers (02), MOOC instructors (03), ICT engineers specialized in education (02), and learners with MOOC experience (03). Following recommendations from [28], the panel size met the accepted range of 5 to 20 participants. Selection was based on criteria such as academic qualifications, research experience, and professional engagement in technology-enhanced learning. Each expert had at least five years of professional experience and direct exposure to blended learning environments. Although experts should have rich experience in working with MOOCs, undergraduate students (MOOC learners) were considered experts, as their voices and experience were valuable. Before the rating process, all participants received detailed instructions to ensure a standardized understanding of the scale and indicators. Their diverse expertise helped ensure that the selected indicators reflected both theoretical soundness and real-world practicality. The demographics of selected experts are presented in Table 2.

**Table 2:** Demographics of selected experts.

Expert	Position	Qualification	Experience
Expert 1	Director of English language program	Ph.D	∼ 25 years
Expert 2	Head of MOOC curriculum developer	Ph.D	∼ 20 years
Expert 3	English lecturer	Ph.D	∼ 18 years
Expert 4	English lecturer	Master of TESOL	∼ 10 years
Expert 5	English lecturer	Master of TESOL	∼ 10 years
Expert 6	English curriculum and program developer	Master of Arts	∼ 8 years
Expert 7	English curriculum and program developer	Master of Arts	∼ 10 years
Expert 8	ICT engineer in English education program	Master of Science	∼ 11 years
Expert 9	ICT engineer in English education program	Master of Science	∼ 7 years
Expert 10	English MOOC instructor	Master of TESOL	∼ 11 years
Expert 11	English MOOC instructor	Master of TESOL	∼ 16 years
Expert 12	English MOOC instructor	Master of TESOL	∼ 21 years
Expert 13	English MOOC learner	Undergraduate	2nd year
Expert 14	English MOOC learner	Undergraduate	2nd year
Expert 15	English MOOC learner	Undergraduate	4th year

### Step 3: linguistic scale and fuzzy numbers

A 7-point linguistic scale was used to capture expert opinions, ranging from “extremely not important” to “extremely important.” Each point on the scale was converted into a triangular fuzzy number (TFN) defined by three values: minimum (m1), most likely (m2), and maximum (m3). These values captured the range of opinions and were recorded in Excel for later analysis. This conversion allowed for better modeling of the uncertainty and subjectivity inherent in expert judgment. For example, if an expert rated a sub-indicator as “important,” it might be represented as the fuzzy number (0.5, 0.7, 0.9). The use of fuzzy numbers helped reduce the rigidity of traditional Likert scales, offering a more nuanced picture of expert consensus. The linguistic scale is presented in Table 3.

**Table 3:** Linguistics scale for the questionnaire.

7-point linguistic scale	Fuzzy scale (m1, m2, m3)
1. extremely not important	0.00, 0.00, 0.10
2. not important	0.00, 0.10, 0.30
3. somewhat not important	0.10, 0.30, 0.50
4. neutral	0.30, 0.50, 0.70
5. somewhat important	0.50, 0.70, 0.90
6. important	0.70, 0.90, 1.00
7. extremely important	0.90, 1.00, 1.00

### Step 4: data collection and averaging

Experts rated each sub-indicator using the 7-point scale. The average fuzzy values (m1, m2, m3) across all responses were computed. To calculate the threshold (d), each expert’s response was compared to the overall average using a standard formula involving differences between individual and average TFNs. This step ensured that each expert’s input was fairly represented while identifying possible outliers. The calculated averages were entered into an Excel spreadsheet specifically designed for fuzzy calculations. Consistency checks were performed to verify there were no missing data or input errors, ensuring the integrity of the dataset before moving to the next phase. The equation is as follows:

$$d\left(\tilde{m}\tilde{n}\right)=\sqrt{\frac{1}{3}\left[{\left(m_{1}-m_{1}\right)}^{2}+{\left(m_{2}-m_{2}\right)}^{2}+{\left(m_{3}-m_{3}\right)}^{2}\right]}$$

In the equation, “n” represents the number of experts participating in the evaluation process and “m” is the fuzzy scale. Fig. 1 describes the examples of examining the d value for IS1 and I20, following the above formula Fig. 1a.

**Fig. 1a:**
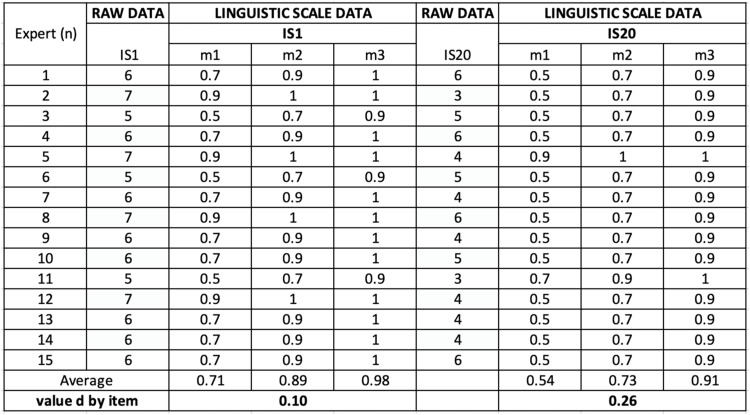
Extract for determining the d-value (threshold value).

### Step 5: threshold value and consensus

The consensus threshold was determined using the value "d". The threshold value (d) used in this study represents the average distance between each expert’s fuzzy value and the aggregated group fuzzy value. This approach follows the standard FDM protocol [27]. The cutoff point of d-value is 0.20 (*d* ≤ 0.2), so the items with a d-value ≤ 0.2 were considered to meet consensus [29]. In addition, a consensus level of at least 75 % was required for a sub-indicator to be retained [30]. The consensus was calculated based on the percentage of experts whose responses met the acceptance threshold or cutoff point (*d* ≤ 0.2). The formula is presented as:

Consensus ( %) = (number of experts (with *d* ≤ 0.2) /total number of experts) x 100

**Fig. 2:**
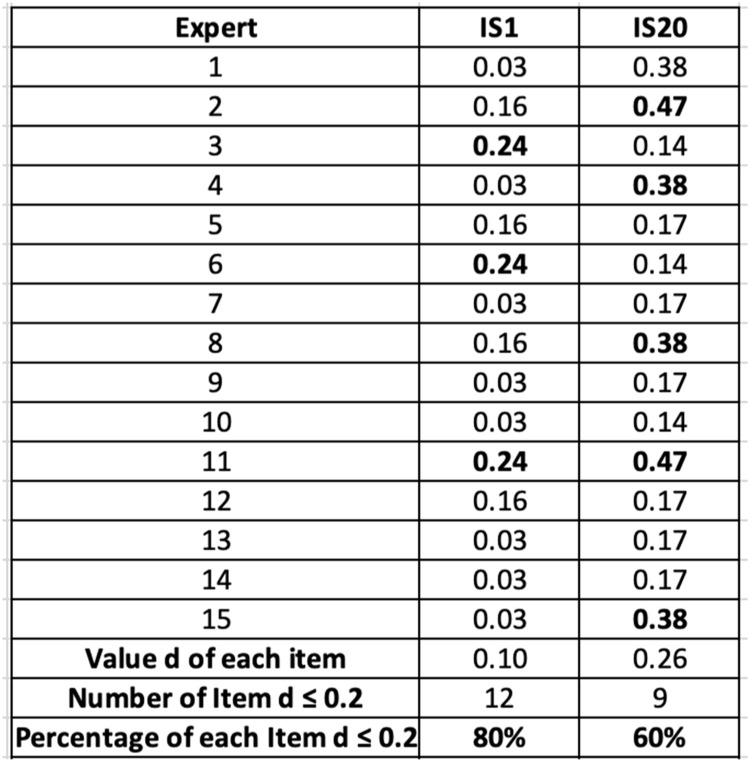
Extract for determining expert approval by items.

This dual criterion enhanced the reliability of the selected indicators. If a sub-indicator did not meet both criteria, additional rounds of evaluation would have been necessary. However, in this study, all retained sub-indicators met both the consensus threshold and agreement level in the first round, indicating strong alignment among experts. Fig. 2 presents the examples of determining expert approval for IS1 and IS20. The results from Fig. 2 indicate that IS1 met the expert consensus at 80 %, while IS20 was at 60 %, based on a cutoff value (*d* ≤ 0.2).

### Step 6: aggregating fuzzy evaluations

The fuzzy responses for each sub-indicator were aggregated to produce a single fuzzy value. This process helped summarize the collective expert opinion across all dimensions of the sub-indicator. This step involved the summation and averaging of all individual TFNs per sub-indicator to arrive at a group-based fuzzy number. The aggregation provided a comprehensive view of importance based on collective judgment. This also eliminated the potential bias from individual outliers, allowing for a balanced interpretation of expert agreement across different roles and disciplines. Specifically, fuzzy aggregation was performed using the fuzzy arithmetic mean of the triangular fuzzy numbers (TFNs). For each sub-indicator, the values m1, m2, and m3 were separately averaged across all expert responses to form the collective TFN. For example, if three experts rated a sub-indicator with fuzzy values (0.5, 0.7, 0.9), (0.4, 0.6, 0.8), and (0.6, 0.8, 1.0), the aggregated TFN would be calculated as the average of each position: m1 = (0.5 + 0.4 + 0.6)/3 = 0.5, m2 = (0.7 + 0.6 + 0.8)/3 = 0.7, and m3 = (0.9 + 0.8 + 1.0)/3 = 0.9. Thus, the collective fuzzy value for this indicator is (0.5, 0.7, 0.9).

### Step 7: Defuzzification

Next, fuzzy values were converted into precise numerical scores through defuzzification. This calculation used the formula:

A=(m1+m2+m3)/3

**Fig. 3:**
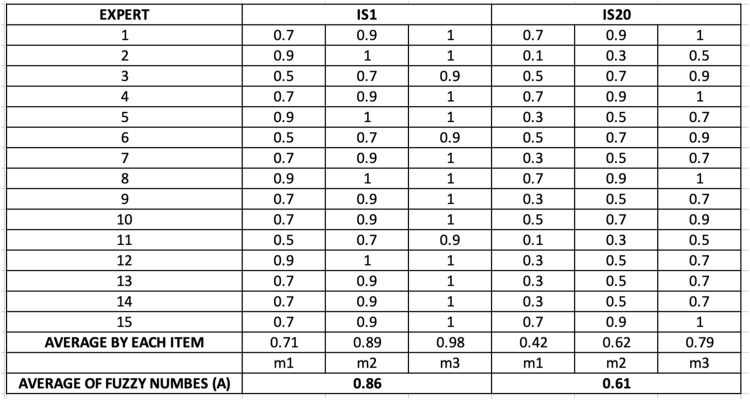
Extract for determining the defuzzified values (A).

While more sophisticated defuzzification methods like the centroid method exist, the simple average formula was employed due to its interpretability, and it is easy to calculate and interpret, widely used in education and social sciences where transparency is prioritized [27]. Specifically, the “A” scores closer to 1 indicated a stronger consensus. Values between 0.00–0.50 reflected low agreement, 0.51–0.74 indicated moderate agreement, and 0.75–1.00 showed strong consensus. Only indicators with high scores were retained in the final list. This transformation allowed for straightforward ranking and comparison of all indicators. The defuzzified scores were entered into a table for analysis and used as a reference for validating the importance of each indicator. Any borderline sub-indicators were flagged for further expert review in case future revisions were required. Fig. 3 describes the examples for defuzzified values (A) for IS1 and IS20. Data from Fig. 3 indicates that experts had a strong consensus on IS1 and a moderate consensus on IS20.

### Step 8: ranking indicators

Finally, the defuzzified values were ranked to determine the relative importance of each indicator. The ranking helped identify which sub-indicators received the most consistent expert endorsement for inclusion in the bMOOC implementation framework for English learning. This prioritization guided the final framework design, focusing resources and attention on the highest-ranked indicators. A ranked list provided clarity on which factors were considered essential by the expert panel. The top five indicators, for example, were proposed as core components of the model, while lower-ranked sub-indicators served as supplementary recommendations.

The methodological steps outlined above provided the basis for analyzing expert consensus on blended MOOC implementation in English language learning, which is detailed in the subsequent section on method validation.

### Method validation

**Fig. 4:**
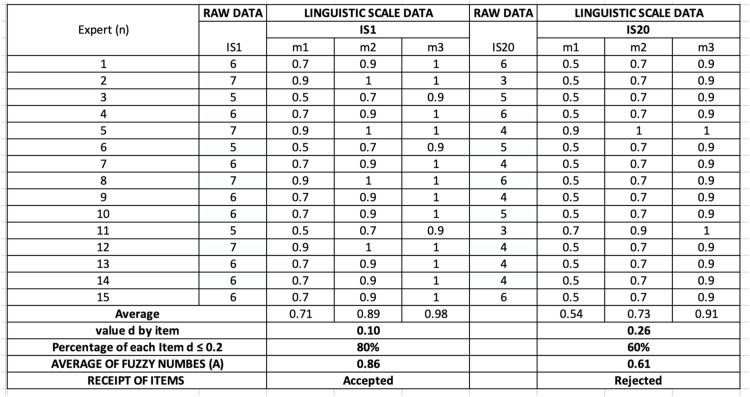
Extract of validating sub-indicators for implementing bMOOCs.

This study utilized the Fuzzy Delphi Method (FDM) to identify and validate essential indicators for implementing blended MOOCs (bMOOCs) in English language learning. The results were analyzed through multiple FDM steps, including threshold computation, consensus evaluation, defuzzification, and ranking. To validate the indicators and sub-indicators, three criteria were applied: 1) threshold *d* ≤ 0.20, 2) experts’ consensus ≥ 75 %, and 3) defuzzified score *A* ≥ 0.50. An example of the validating sub-indicators for bMOOCs implementation is described in Fig. 4 To examine the robustness of the FDM results, a sensitivity analysis was performed by varying the cutoff thresholds. The d-value cutoff was tested at 0.15, 0.20, and 0.25; the consensus percentage was tested at 70 %, 75 %, and 80 %; and the threshold value was tested at 0.45, 0.50, and 0.55. The results indicated that the final set of accepted indicators remained stable under these variations, confirming its robustness. The final validated sub-indicators comprise 20 sub-indicators categorized under six thematic domains: learner analysis, instructional objectives, content and technology, teaching strategies, learner engagement, and assessment and feedback.

### Threshold value (d) analysis

The threshold value (d) is a critical indicator of expert consensus. According to FDM standards, a d-value of 0.2 or below signifies sufficient agreement among experts. From the data analysis, 20 sub-indicators met the threshold requirement, with d-values ranging from 0.03 to 0.19, which is lower than the cutoff point (*d* ≤ 0.20). These low values indicate strong alignment in expert judgments, affirming the clarity, importance, and appropriateness of the indicators for bMOOC implementation in English language contexts. Only one sub-indicator SI20 was rejected as the d-value was 0.26, which exceeded the accepted range.

The sub-indicator with the lowest d-value was SI8 (*d* = 0.03), reflecting exceptionally high agreement among panelists. Conversely, SI14 registered the highest d-value (*d* = 0.19), though still within acceptable limits, suggesting moderate consensus. Notably, the average threshold value across all sub-indicators was 0.13, demonstrating that overall, expert agreement was robust and within methodological standards.

#### Consensus level evaluation

Expert agreement was further evaluated by calculating the percentage of respondents who agreed on each sub-indicator’s inclusion. A minimum consensus level of 75 % was required. All accepted sub-indicators exceeded this threshold, with three sub-indicators achieving 100 % consensus, notably SI3, SI12, and SI13. These results indicate very high alignment in expert perceptions of core components needed for effective bMOOC implementation for English learning.

Other indicators received the minimum acceptable consensus of 80 % (except SI20). Although they met the basic criteria, the relatively lower agreement suggests they might be more context-dependent or require clearer definitions in future applications. The only rejected indicator, SI20, received the lowest consensus at 60 %, further justifying its exclusion.

#### Defuzzification and linguistic ranking

To convert expert judgments from fuzzy values into precise scores, defuzzification was conducted using the formula *A* = (m1 + m2 + m3)/3. The defuzzified scores ranged from 0.61 to 0.89, showing varying levels of perceived importance across the indicators.

The sub-indicators were ranked into three categories based on defuzzified values:

*- High importance (A ≥ 0.83): SI1, SI3, SI4, SI5, SI8, SI9, SI13*

Indicators in this group were viewed as the most essential for successful bMOOC implementation. They received the strongest support from experts and reflect the core components of effective online English learning environments:

- Moderate importance (*A* = 0.75 to 0.83): SI6, SI7, SI10, SI11, SI12, SI14, SI15, SI16, SI17, SI18, SI19

This group includes indicators that were still considered important but with slightly lower consensus. These factors are supportive in nature and contribute to enhancing the teaching and learning experience:

*- Low importance (A < 0.75): SI2, SI21*

The sub-indicators in this group scored lower, indicating weaker consensus on their criticality. While still above the inclusion threshold, they may be considered context-specific or less central to the framework.

One indicator, SI20 (*A* = 0.61), was the lowest-rated and did not meet the required threshold or consensus criteria. It was therefore excluded from the final list of validated indicators.

#### Key indicators validation

The validation results confirmed six key indicators for blended MOOC implementation in English language learning. All indicators achieved threshold values below 0.2, indicating strong experts’ agreement. The highest-ranked indicator was “analysis of learners” (*A* = 0.830), followed by “course materials and technology” (*A* = 0.828) and “objectives” (*A* = 0.82). Although ranked lower, Teaching methodologies, “learners’ participation”, and “feedback and assessment” also met the validation criteria, ensuring a well-rounded framework grounded in both pedagogical and practical priorities.

### Limitations, implications, and recommendations

While the Fuzzy Delphi Method (FDM) provides a structured and systematic approach for reaching expert consensus, several limitations should be acknowledged. First, the sample size of 15 experts, though within the recommended range, may limit the generalizability of findings across broader educational contexts. Second, the fuzzy scales used rely on subjective interpretation, and despite efforts to standardize understanding through detailed instructions, variations in expert judgment could still affect the results. Additionally, the study carried out several rounds of evaluation; while a strong consensus was reached, more rounds might have provided deeper insights or improved the indicators. Lastly, the indicators were validated within the context of higher education in Vietnam and may need to be adapted for use elsewhere.

The study offers several practical implications in the field of bMOOCs and EFL. Firstly, instructional designers can use the validated indicators to align MOOC content with in-class activities, ensuring a coherent blended experience. Policymakers may also incorporate these indicators into national quality assurance frameworks to standardize digital language instruction. Additionally, teacher training programs can embed these indicators as benchmarks for lesson planning and technology integration. Future studies should be conducted in different regions and educational levels to test its adaptability, and it would be convenient to carry out a comparative study with students. Longitudinal studies could also examine how these indicators influence actual learning outcomes over time.

## Ethics statements

This study was conducted in line with established ethical standards for educational research. Before data collection, informed consent was obtained from all participating teachers, and formal approval was granted by the institution involved. To ensure confidentiality, all data were anonymized, and participants were aware of their right to withdraw from the study at any stage without any negative consequences.

## CRediT authorship contribution statement

**Anh Tuan Pham:** Writing – review & editing, Writing – original draft, Visualization, Validation, Supervision, Software, Resources, Project administration, Methodology, Investigation, Funding acquisition, Formal analysis, Data curation, Conceptualization. **Muhammad Ridhuan Tony Lim Abdullah:** Writing – review & editing, Writing – original draft, Visualization, Validation, Supervision, Software, Resources, Project administration, Methodology, Investigation, Funding acquisition, Formal analysis, Data curation, Conceptualization.

## Declaration of competing interest

The authors declare that they have no known competing financial interests or personal relationships that could have appeared to influence the work reported in this paper.

## Data Availability

Data will be made available on request.
